# Susceptibility and Multidrug Resistance Patterns of *Escherichia coli* Isolated from Cloacal Swabs of Live Broiler Chickens in Bangladesh

**DOI:** 10.3390/pathogens8030118

**Published:** 2019-07-31

**Authors:** Muha. Ajijur Rahman Al Azad, Md. Masudur Rahman, Ruhul Amin, Mst. Ismat Ara Begum, Reinhard Fries, Asmaul Husna, Ahmed S. Khairalla, A.T.M. Badruzzaman, Mohamed E. El Zowalaty, Kannika Na Lampang, Hossam M. Ashour, Hafez Mohamed Hafez

**Affiliations:** 1 Department of Veterinary Biosciences and Veterinary Public Health, Faculty of Veterinary Medicine, Chiang Mai University, Chiang Mai 50100, Thailand; 2 Department of Pathology, Faculty of Veterinary, Animal and Biomedical Sciences, Sylhet Agricultural University, Sylhet 3100, Bangladesh; 3 Bangladesh Council for Scientific and Industrial Research, Rajshahi 6206, Bangladesh; 4 Department of Animal Husbandry and Veterinary Science, Rajshahi University, Rajshahi 6205, Bangladesh; 5 Institute of Food Safety and Food Hygiene, Freie Universitaet Berlin, 14195 Berlin, Germany; 6 Department of Microbiology & Immunology, Faculty of Pharmacy, Beni-Suef University, Beni-Suef 62511, Egypt; 7 Virology & Microbiology Research Group, College of Pharmacy, City University College of Ajman, Al Tallah 2, Ajman P.O. Box 18484, UAE; 8 Department of Biological Sciences, College of Arts and Sciences, University of South Florida St. Petersburg, St. Petersburg, FL 33701, USA; 9 Department of Microbiology and Immunology, Faculty of Pharmacy, Cairo University, Cairo 11562, Egypt; 10 Institute of Poultry Diseases, Faculty of Veterinary Medicine, Freie Universitaet Berlin, 14195 Berlin, Germany

**Keywords:** *E. coli*, antibiotics, multi-drug resistance, broiler chicken, zoonotic, Bangladesh

## Abstract

Antimicrobial resistance is a major health problem, particularly in developing countries like Bangladesh, where there is a paucity of information on resistance patterns and prevalence of antimicrobial determinants. Therefore, the aims of this study were to investigate the prevalence of resistance, including multi-drug resistance (MDR), and the associated genetic determinants in *Escherichia coli* isolates from cloacal swabs of live broiler chickens in Bangladesh. Altogether, 400 cloacal swabs (200 from Rajshahi and 200 from Dhaka divisions) were randomly collected from individual chickens in 50 broiler farms. *E. coli* was isolated and identified using conventional bacteriological culture and biochemical methods. The isolates were further confirmed using genus-specific 16S rRNA-targeted polymerase chain reaction (PCR) primers. Antimicrobial susceptibilities and MDR of the isolates against nine different antimicrobial agents (ampicillin, erythromycin, tetracycline, gentamicin, ciprofloxacin, levofloxacin, trimethoprim-sulfamethoxazole, colistin sulphate, and streptomycin) were determined using the Kirby-Bauer disc diffusion method. Resistance determinants of *E. coli* to ampicillin (*bla*_TEM_), streptomycin (*aadA1*), erythromycin [*ere*(A)], trimethoprim (*dfrA1*), and tetracycline [*tet*(A), *tet*(B)] were screened using PCR. Our results showed that all swab samples were positive for *E. coli.* The isolates were uniformly resistant to ampicillin, tetracycline, streptomycin, ciprofloxacin, erythromycin, and trimethoprim-sulphamethoxazole. The isolates exhibited highest susceptibility to colistin sulphate (73.5%), followed by gentamicin (49%), and levofloxacin (17%). All isolates were resistant to three classes of antibiotics, 204 isolates (51%) were resistant to four classes, and 56 isolates (14%) were resistant to five. The highest prevalence of antimicrobial resistance gene was recorded for tetracycline (*tet*(A):95.25%; *tet*(B):95.25%) followed by ampicillin (*bla*_TEM_:91.25%), streptomycin (*aadA1*:88.25%), erythromycin (*ere*(A):84.75%), and trimethoprim (*dfrA1*:65.5%). In conclusion, surveillance for MDR bacteria in poultry is a critical piece of knowledge, which would be useful for optimizing empiric antimicrobial treatments and exploring alternative antimicrobial agents.

## 1. Introduction

Antimicrobial resistance, which is caused mainly by the imprudent use of antimicrobial agents [1,2,3], is becoming an increasing global concern in animals and humans. Due to the magnitude of the threat, the World Health Organization (WHO) recommended global surveillance programs in animal and human populations

*Escherichia (E.) coli* is a common microbial inhabitant of the gastrointestinal tract of poultry, humans, and other animals [4]. While most strains of *E. coli* are nonpathogenic and may serve as indicators of fecal contamination of food and water, up to 15% of intestinal coliforms can be opportunistic and pathogenic in nature [5]. Pathogenic *E. coli* negatively impacts immunocompromised hosts and poultry, causing severe diseases such as meningitis, endocarditis, urinary tract infections, septicemia, and epidemic diarrhea [6]. Other diseases include yolk sac infection, omphalitis, cellulitis, swollen head syndrome, coligranuloma, and colibacillosis in poultry [7].

Improper antimicrobial treatment is the main culprit that promotes the emergence, selection, and spread of antimicrobial resistant bacterial strains among animals and humans [8,9]. The zoonotic spread of antimicrobial resistance has been previously reported [10,11,12,13]. Emergence of multi-drug resistance (MDR) to antimicrobial agents may lead to increased morbidity, mortality, and healthcare costs [14].

The poultry production industry in Bangladesh is an important source of income, with estimates around 170 million broilers and layer chickens being produced from more than 115,000 farms, according to a recent report from the Department of Livestock Services (DLS). Unfortunately, antimicrobial agents are extensively used for the prevention and/or treatment of diseases of food animals. Furthermore, the extensive use of antimicrobial agents in humans has exacerbated the spread of resistance [3]. There is a paucity of information in the literature on MDR in poultry in Bangladesh. The present study was conducted to isolate *E. coli* strains from live broiler chickens in Bangladesh, in order to determine their susceptibility and MDR patterns to selected antimicrobial agents commonly used in both food animals and humans.

## 2. Material and Methods

### 2.1. Ethics Statement

The current study was conducted at the Department of Pathology, Faculty of Veterinary, Animal and Biomedical Sciences, Sylhet Agricultural University, Bangladesh, as well as the Department of Veterinary Public Health and Bio Science, Faculty of Veterinary Medicine, Chiang Mai University, Thailand. The handling of animals in this study was performed in accordance with the current Bangladesh legislation (Cruelty to Animals Act 1920, Act No. I of 1920 of the Government of the People’s Republic of Bangladesh). In addition, all our procedures for animal experiments were approved by the Ethics Committee of the Sylhet Agricultural University, Bangladesh and Chiang Mai University, Thailand.

### 2.2. Sample Collection

A total of 400 cloacal swabs, 200 from Rajshahi division and 200 from Dhaka division, was randomly collected from individual broiler chickens from 50 different broiler farms. Farms were selected from the list of broiler farms available at the Department of Livestock Services, Dhaka Bangladesh by a multistage random selection method. All farms that have been running for at least two years with at least 500 broilers in each batch were considered for sampling. Eight broilers were randomly selected from the same farm and cloacal swabs were collected aseptically using sterile cotton swabs and kept separately in sterile tubes. After collection, the samples were immediately transported to the laboratory under cold conditions for further processing.

### 2.3. Isolation and Identification of E. coli

On the day of arrival, cloacal swabs were streaked on MacConkey agar (Merck, Darmstadt, Germany) and incubated aerobically at 37 °C for 24 h. Lactose-fermenting colonies were then picked and re-streaked on eosin methylene blue (EMB) agar (Merck, Darmstadt, Germany) and incubated for 24 h at 37 °C. The green metallic sheen colonies were considered to be *E. coli*. These colonies were further tested biochemically, including the growth on triple sugar iron agar (TSI) and lysine iron agar (LIA), the oxidative/fermentative metabolism of glucose, as well as the abilities for citrate utilization, urease production, indole fermentation, tryptophan degradation, glucose degradation (methyl red test), and motility. *E. coli* isolates were stored in tryptic soy broth (Merck, Darmstadt, Germany) containing 15% glycerol at −20 °C [15]. Molecular confirmation of the isolates was performed using PCR targeting the 16S rRNA, using a primer set specific for *E. coli*, as previously described [16].

### 2.4. Antimicrobial Susceptibility Testing by Disc Diffusion Method

Antimicrobial susceptibility testing of *E. coli* isolates was performed on Muller-Hinton agar plates (Merck, Darmstadt, Germany) using the Kirby-Bauer disc diffusion method according to the Clinical and Laboratory Standards Institute (CLSI) guidelines [17]. The antimicrobial agents tested (Oxoid, England), included ampicillin (10 µg), colistin (10 µg), gentamicin (10 µg), streptomycin (10 µg), ciprofloxacin (5 µg), levofloxacin (5 µg), erythromycin (15 µg), trimethoprim-sulfamethoxazole (1.25/23.75 µg), tetracycline (30 µg). These antimicrobial agents were selected since they are the most commonly used agents in broiler farms in Bangladesh and owing to their public health relevance, as recommended by the CLSI. A standard strain of *E. coli* (ATCC 25922; American Type Culture collection, Rockville, MD, USA) was used as a control in all experiments. The susceptibilities of *E. coli* isolates to individual antimicrobial agents were determined and interpreted following aerobic incubation at 37 °C for 18 to 24 h, according to CLSI guidelines. Test results were only considered valid when the diameters of the inhibition zones of the control *E. coli* (ATCC 25922) strain were within the performance ranges. Resistant and intermediate resistant isolates were collectively referred to as non-susceptible, as previously described [18]. Isolates were considered as multidrug resistant when found non-susceptible to at least one agent in three or more antimicrobial different classes of antimicrobial agents, excluding the broad-spectrum penicillins without a β-lactamase inhibitor [18].

### 2.5. Detection of Selected Antimicrobial Resistance Genes by PCR Assay

*E. coli* isolates were sub-cultured overnight in Luria-Bertani broth (Merck, Darmstadt, Germany) and their genomic DNA was extracted using a genomic DNA extraction kit (Fermentas, Germany) according to the manufacturer’s instructions. The concentration and purity of the eluted DNA were determined using the NanoDrop^™^ 2000c (Thermo Scientific, Waltham, MA, USA). The extracted DNA was then subjected to a PCR to screen for the presence of six genes in *E. coli* that have been associated with resistance to ampicillin (*bla*_TEM_), streptomycin (*aadA1*), erythromycin [*ere*(A)], trimethoprim (*dfrA1*) and tetracycline [*tet*(A), *tet*(B)]. The set of primers used for each gene [19,20,21,22] and the amplicon sizes are shown in Table 1. The PCR reactions, conducted in a Gene Cycler (Bio-Rad, Hercules, CA, USA), were performed in a final volume of 25 µL containing 1× reaction buffer (Promega, Madison, Wl, USA), 2.5 U of *Taq* polymerase (Promega, Madison, Wl, USA), 0.2 mM each deoxynucleotide triphosphate (dNTP), 1.5 mM Magnesium chloride, 20 pmol of each primer, and 5 µL (40–260 ng/µL) of extracted DNA as a template. Each PCR amplification cycle consisted of an initial denaturation step at 95 °C for 10 min, followed by denaturation at 95 °C for 30s, annealing at 50 or 58 °C (depending on primers used, Table 1) for 30s, and extension at 72 °C for 1 min for each kb of DNA amplified. This cycle was repeated 30 times followed by a final extension step at 72 °C for 10 min. The amplified PCR products were electrophoresed and visualized using 1.5% agarose gels and amplicons were photographed using a gel documentation system (Uvitec, UK). A molecular weight marker with 100 bp increments (100 bp DNA ladder, Invitrogen^™^, Massachusetts, USA) was used as a size standard. Genomic DNA extracted from *E. coli* O157:K88ac:H19, CAPM 5933, O159:H20, and CAPM 6006 strains was used as positive controls, while reactions to which no template DNA has been added served as our negative control.

## 3. Results

All the 400 specimens collected in the current study were positive for *E. coli* based on the conducted microbiological and biochemical tests (Table 2). The identity of these isolates was further supported by the PCR-positive results obtained using a published *E. coli* 16S rRNA gene primer set [16].

Antimicrobial resistance and susceptibility patterns of the tested *E. coli* isolates against nine selected antimicrobial agents were determined using the agar disc diffusion method; the results are summarized in Table 3. All the isolates (100%) were resistant to six antimicrobial agents, namely ampicillin, tetracycline, streptomycin, ciprofloxacin, erythromycin, and trimethoprim-sulphamethoxazole. On the other hand, the highest susceptibility rates were recorded against colistin sulphate (73.5%), followed by gentamicin (49%), and levofloxacin (17%) (Table 3).

The MDR patterns of the tested *E. coli* isolates were also evaluated against five different antimicrobial classes (Table 4). All the isolates (100%) showed MDR against 3 antimicrobial classes (tetracycline, trimethoprim-sulphamethoxazole, and ciprofloxacin). Out of the 400 *E. coli* isolates, 204 (51%) were resistant to four antimicrobial classes, while 56 (14%) were resistant to five classes (Table 4).

Finally, the isolates in the current study were PCR-screened for the presence of six selected antimicrobial resistance genes, including those associated with resistance to ampicillin (*bla*_TEM_), streptomycin (*aadA1*), erythromycin [*ere*(A)], trimethoprim (*dfrA1*) and tetracycline [*tet*(A), *tet*(B)]. The highest prevalence among these genes was recorded for tetracycline (*tet*(A): 95.25%; *tet*(B): 95.25%) followed by ampicillin (*bla*_TEM_:91.25%), streptomycin (*aadA1*:88.25%), erythromycin (*ere*(A):84.75%), and trimethoprim (*dfrA1*:65.5%) (Table 5).

## 4. Discussion

Emergence of bacterial resistance to antimicrobial agents has become a significant and prevalent public health threat especially when there are few or no available alternative effective antimicrobial agents for the treatment of infections caused by these bacteria. Though most strains of *E. coli* are harmless and commonly found in the gut of humans and warm-blooded animals, some strains can cause severe foodborne illness in humans.

In the present study, we investigated the prevalence and the determinants of antibiotic resistance among *E. coli* isolates recovered from cloacal swabs of broiler chickens in Bangladesh. The 100% prevalence of *E. coli* among the samples collected in the current study is higher than several previous reports from Bangladesh [24,25,26]. Whereas previous studies in Bangladesh reported the isolation and identification of *E. coli* through conventional bacteriological and biochemical tests, the present study included an additional PCR-based molecular confirmatory test that used a previously published *E. coli*-specific 16S rRNA primer set [16].

Antimicrobial resistance in chickens is a common problem in Bangladesh and other developing countries due to the indiscriminate use of antibiotics as feed additives and prophylactic treatment of infectious diseases. According to our study, all isolates of *E. coli* were resistant in varying degrees to commonly used antimicrobial agents, such as ampicillin, tetracycline, streptomycin, ciprofloxacin, erythromycin, trimethoprim-sulphamethoxazole, colistin sulphate, gentamicin, and levofloxacin. Our resistance rates were higher than most of those reported in previous studies. For instance, the resistance rates reported in Bangladesh by Hossain et al. [26] were 91.42% for erythromycin and 62.85% for ampicillin. Similarly, in Ethiopia, 90%, 78%, and 60% of *E. coli* isolates were resistant to tetracycline, streptomycin, and ampicillin, respectively [27]. All the isolates (100%) in our present study were resistant to the previous list of antibiotics. In another study conducted in Bangladesh, Hashem and colleagues [28] reported *E. coli* isolates that were 100% susceptible to colistin sulphate. The rate of susceptibility was only 73.5% among our isolates. This can reflect either the abuse of colistin sulphate, or the acquisition of colistin resistance genes while integrating other antibiotic resistance genes, if these determinants are located on the same mobile genetic element. One of the closest finding to our results was the findings of Al-Ghamdi et al. [29] who showed a very high resistance level of *E. coli* isolates (99.1%) to tetracycline in Saudi Arabia. 

The emergence of *E. coli* isolates with varying MDR phenotypes, involving co-resistance to three or more different antibiotic classes, has been previously reported and is now considered to be an escalating public health issue [30,31,32]. Similar to previous reports [33,34], the current study exhibited high resistance rates to different classes of antimicrobial agents. More specifically, the *E. coli* isolates in the present study were found to be multi-resistant to several commonly used antimicrobial agents (including tetracycline, trimethoprim-sulphamethoxazole, ciprofloxacin, gentamicin, and colistin sulphate). All *E. coli* isolates exhibited MDR to more than three antimicrobial agents of different families. Similar findings on MDR patterns of *E. coli* isolates have been reported in Bangladesh and other parts of the world [12,13,29,35]. A relatively lower prevalence of MDR *E. coli* was reported in broiler chicken in neighboring countries, such as India (94%) [36] and Nepal (80.0%) [37]. However, up to our knowledge, most previous studies on MDR of *E. coli* did not follow the recent recommendations of MDR determination made by the European Centre for Disease Prevention and Control (ECDC) and the Centers for Disease Control and Prevention [23]. The higher MDR among *E. coli* isolates in the present study can be due to the unnecessary overuse of antimicrobial agents as feed additives or prophylactic treatments in chickens. According to our survey, 80% of poultry farmers in Bangladesh used antimicrobial agents as preventive treatment (data not shown). It is alarming that poultry farmers are practicing imprudent administration of antimicrobial agents. The most common antimicrobial agents used by the farmers in the studied areas included ciprofloxacin, enrofloxacin, levofloxacin, doxycycline, trimethoprim-sulphamethoxazole, and colistin sulphate. Antimicrobial agents are accessible and can be purchased without prescription from veterinarians in Bangladesh. Due to the excessive use of antimicrobial agents, microorganisms with MDR may ultimately replace drug-sensitive microorganisms in environments saturated with antimicrobial agents [38].

In the current study, we also screened the isolates for the presence of selected antimicrobial resistance genes, including those for ampicillin (*bla*_TEM_), streptomycin (*aadA1*), erythromycin [*ere*(A)], trimethoprim (*dfrA1*), and tetracycline [*tet*(A), *tet*(B)]. The prevalence of these genes was generally higher in the present study than in previous studies [39,40]. This may explain the relatively high rates of resistance to those antibiotics in the present study, as assessed by the disc diffusion method.

In conclusion, the relatively high MDR levels among *E. coli* isolates in the current study can be attributed to the excessive use of antimicrobial agents in Bangladesh. In this regard, it has to be highlighted that only farms from 2 divisions out of 8 known divisions in Bangladesh were studied. Therefore, our results may not completely reflect the prevalence of MDR *E. coli* strains in the country as a whole. Strict guidelines for the use of antimicrobial agents in food animals and comprehensive antimicrobial drug administration monitoring systems should be urgently advocated and implemented, especially in developing countries like Bangladesh, to reduce the emergence of MDR. In addition, further research on alternative antimicrobial agents to current antibiotics is urgently needed to compensate for the shortage of effective antibiotics.

## Figures and Tables

**Table 1:** PCR primers used in this study for the screening of antimicrobial resistance genes in the tested *E. coli isolates*.

Antimicrobial Agent	Resistance Gene	Primer Sequence (5′→3′ Direction) ^§^	Amplicon Size (bp)	Annealing Temperature (°C)	Reference
Ampicillin	*bla*_TEM_	(F) TGG GTG CAC GAG TGG GTT AC	526	58	[21]
		(R) TTA TCC GCC TCC ATC CAG TC			
Streptomycin	*aadA1*	(F) TAT CCA GCT AAG CGC GAA CT	447	58	[22]
		(R) ATT TGC CGA CTA CCT TGG TC			
Erythromycin	*ere*(A)	(F) GCC GGT GCT CAT GAA CTT GAG	419	58	[22]
		(R) CGA CTC TAT TCG ATC AGA GGC			
Trimethoprim	*dfrA1*	(F) GGA GTG CCA AAG GTG AAC AGC	367	58	[20]
		(R) GAG GCG AAG TCT TGG GTA AAA AC			
Tetracycline	*tet*(A)	(F) GGT TCA CTC GAA CGA CGT CA	577	50	[19]
		(R) CTG TCC GAC AAG TTG CAT GA			
	*tet*(B)	(F) CCT CAG CTT CTC AAC GCG TG	634	50	[19]
		(R) GCA CCT TGC TGA TGA CTC TT			

^§^ (F): forward primer; (R): reverse primer.

**Table 2:** Distribution of the recovered isolates as a function of sampling location.

Sampling Locations	No. of Farms Investigated	No. of Samples Taken ^§^	No. of *E. coli* Isolates Recovered
Dhaka division	25	200	200
Rajshahi division	25	200	200
Overall	50	400	400

^§^ Eight cloacal swab samples from individual chickens were collected from each farm. The identities of the isolates were confirmed by PCR using *E. coli*-specific 16S rRNA primer set.

**Table 3:** Antimicrobial susceptibility patterns among the investigated *E. coli* isolates in relation to the geographical location of the tested broiler farms.

Antimicrobial Agents ^§^	No. (%) of *E. coli* Isolates					
	Rajshahi (*n* = 200)		Dhaka (*n* = 200)		Overall (*n* = 400)	
	Susceptible	Non-Susceptible	Susceptible	Non-Susceptible	Susceptible	Non-Susceptible
Colistin	160 (80)	40 (20)	134 (67)	66 (33)	294 (73.5)	106 (26.5)
Gentamicin	102 (51)	98 (49)	94 (47)	106 (53)	196 (49.0)	204 (51.0)
Levofloxacin	53 (26.5)	147 (73.5)	15 (7.5)	185 (92.5)	68 (17.0)	332 (83.0)

^§^ All the tested *E. coli* isolates (whether from Rajshahi or Dhaka divisions) were fully resistant to ampicillin, tetracycline, streptomycin, ciprofloxacin, erythromycin, and trimethoprim-sulphamethoxazole. Resistant and intermediately-resistant isolates are collectively referred to as non-susceptible.

**Table 4:** MDR patterns among the investigated *E. coli* isolates in relation to the geographical location of the tested broiler farms.

No. of Antibiotic Classes to Which the Tested Strain Exhibits Resistance (*n* = 5)	Antimicrobial Agents ^§^	No. (%) of *E. coli* Isolates Exhibiting MDR		
		Rajshahi (*n* = 200)	Dhaka (*n* =200)	Overall (*n* = 400)
3	TE + TS + CIP	200 (100)	200 (100)	400 (100)
4	TE + TS + CIP + GE	100 (50)	104 (52)	204 (51)
5	TE + TS + CIP + GE + COL	25 (12.5)	31 (15.5)	56 (14)

^§^ TE = Tetracycline, TS = Trimethoprim-sulfamethoxazole, CIP = Ciprofloxacin, GE = Gentamicin, COL = colistin. MDR was determined according to the recommendations by the European Centre for Disease Prevention and Control (ECDC) and the Centers for Disease Control and Prevention [23].

**Table 5:** Prevalence of antibiotic resistance genes among the investigated *E. coli* isolates in relation to the geographical location of the tested broiler farms.

Division	No. (%) of *E. coli* Isolates with Positive Results for Antibiotic Resistance Genes ^§^					
	*bla*_TEM_	*aadA1*	*ere*(A)	*dfrA1*	*tet*(A)	*tet*(B)
Rajshahi (*n* = 200)	171 (85.5)	155 (77.5)	152 (76)	103 (51.5)	192 (96)	184 (92)
Dhaka (*n* = 200)	194 (97)	198 (99)	187 (93.5)	159 (79.5)	189 (94.5)	197 (98.5)
Overall (*n* = 400)	365 (91.25)	353 (88.25)	339 (84.75)	262 (65.5)	381 (95.25)	381 (95.25)

^§^ The prevalence of genes related to resistance to ampicillin (*bla*_TEM_), streptomycin (*aadA1*), erythromycin [*ere*(A)], trimethoprim (*dfrA1*), and tetracycline [*tet*(A), *tet*(B)] was determined by PCR.
